# Operationalizing trustworthy artificial intelligence in clinical and operational workflows

**DOI:** 10.3389/fdgth.2026.1779041

**Published:** 2026-02-13

**Authors:** Kunal Khashu

**Affiliations:** HCA Healthcare, Nashville, TN, United States

**Keywords:** artificial intelligence (AI), digital health (DH), digital transformation, healthcare administration, responsible AI

## Abstract

Artificial intelligence (AI) is increasingly deployed across healthcare systems to support clinical decision-making, optimize operational processes, and improve population health outcomes. Despite substantial investment and rapid advances in model performance, real-world adoption and sustained impact remain limited. A central barrier is the challenge of trust among clinicians, administrators, patients, and regulators in AI-enabled systems. While the concept of “trustworthy AI” is widely invoked, existing frameworks largely emphasize technical model properties and ethical principles without sufficient guidance for operational implementation. This paper argues that trustworthiness is not an intrinsic attribute of AI models but an emergent property of socio-technical systems in which AI is embedded. We propose a comprehensive framework for operationalizing trustworthy AI that shifts attention from model-centric validation to workflow-level design, governance, and continuous evaluation. By integrating decision-centered design, human–AI role delineation, failure visibility, embedded accountability, and longitudinal performance monitoring, the framework provides a pragmatic foundation for deploying AI systems that are safe, equitable, and sustainable in both clinical and operational contexts.

## 1 Introduction

Artificial intelligence has become a central pillar of contemporary digital health strategy. Applications now span diagnostic imaging, clinical risk prediction, triage, workforce scheduling, capacity management, revenue cycle optimization, and population health surveillance. Numerous studies report impressive algorithmic performance in retrospective evaluations, often exceeding human benchmarks in narrowly defined tasks. Yet, translation of these advances into consistent improvements in patient outcomes, operational efficiency, or clinician experience has been uneven.

This gap between technical promise and real-world impact has prompted increasing scrutiny of how AI systems are designed, deployed, and governed in healthcare settings. Concerns related to bias, transparency, safety, accountability, and unintended consequences have catalyzed the emergence of the concept of *trustworthy AI*. Global organizations, including the World Health Organization (WHO) and international research consortia, have articulated ethical and operational principles intended to guide responsible AI development and deployment (1).

Despite this progress, health systems continue to face practical challenges in translating abstract principles into operational reality. Many AI initiatives fail not because models are inaccurate, but because they are misaligned with clinical workflows, poorly integrated into decision processes, or insufficiently governed once deployed (2). As a result, clinicians may distrust AI outputs, override recommendations inconsistently, or disengage from AI tools altogether. The effectiveness of artificial intelligence in healthcare depends on its ability to augment human expertise rather than replace clinical judgment, requiring careful integration into workflows that preserve professional autonomy and accountability (3).

This paper contends that these challenges stem from an overly narrow, model-centric conception of trustworthiness. We argue that trustworthiness must be understood as a system-level property that emerges from the interaction between AI technologies, human actors, organizational processes, and governance structures. Building on existing guidelines and socio-technical theory, we propose a framework for operationalizing trustworthy AI that emphasizes workflow integration, decision accountability, and continuous oversight across the AI lifecycle.

## 2 The evolution of trustworthy AI in healthcare

### 2.1 From technical performance to ethical principles

Early AI research in healthcare focused primarily on predictive accuracy, sensitivity, and specificity. As models became more complex and influential, concerns emerged regarding bias, explainability, and generalizability. In response, scholars and policymakers advanced ethical principles intended to constrain harmful applications of AI, often drawing on broader discussions of AI ethics across industries.

The WHO's guidance on the ethics and governance of AI for health articulates six core principles, including human autonomy, well-being, transparency, accountability, inclusiveness, and sustainability (1).

These frameworks represent an important shift from narrow technical evaluation toward a more holistic understanding of AI's societal and clinical implications. However, they remain largely aspirational, offering limited direction on how principles should be operationalized within specific workflows or organizational contexts.

### 2.2 Limitations of principle-based approaches

Principle-based frameworks are intentionally broad to accommodate diverse healthcare systems and use cases. Yet this generality also limits their operational utility. Health systems implementing AI must make concrete decisions about workflow integration, user interfaces, escalation pathways, and governance responsibilities; areas that are only indirectly addressed by high-level ethical guidance.

Moreover, principles such as fairness or transparency may conflict in practice. For example, increasing model complexity may improve accuracy but reduce interpretability; aggressive bias mitigation may alter performance across subpopulations in unintended ways. Without explicit mechanisms for managing these trade-offs, health systems are left to navigate trustworthiness challenges on an *ad hoc* basis (4).

These limitations suggest the need for frameworks that bridge ethical aspirations with operational realities, explicitly addressing how AI systems function within everyday clinical and administrative work.

## 3 Trustworthiness as a socio-technical property

### 3.1 A shift in conceptualization

Traditional evaluations of AI trustworthiness focus on properties of the algorithm itself. In contrast, socio-technical theory emphasizes that system behavior arises from the interaction between technical components and social context. From this perspective, an AI model cannot be trustworthy in isolation; its trustworthiness depends on how it is used, interpreted, monitored, and governed.

This framing aligns with empirical observations that trust failures often occur despite technically sound models. For instance, clinicians may distrust accurate models if recommendations conflict with clinical intuition or workflow constraints. Conversely, poorly performing models may be trusted excessively if embedded in authoritative interfaces or institutional mandates.

Explainability has been identified as a necessary but insufficient condition for trustworthy AI, as understanding must be situated within clinical context, professional responsibility, and governance structures (5).

Understanding trustworthiness as emergent shifts analytical focus from pre-deployment validation to ongoing system performance, user behavior, and organizational accountability.

### 3.2 Implications for AI lifecycle management

If trustworthiness is emergent, it cannot be fully established at the point of deployment. Instead, it must be continuously produced through monitoring, feedback, and adaptation. This implies that responsibility for trustworthiness extends beyond data scientists to include clinicians, operational leaders, informatics teams, and governance bodies (6).

Operationalizing trustworthy AI therefore requires explicit attention to workflow design, decision authority, failure management, and longitudinal evaluation; domains often underemphasized in technical AI research.

## 4 A framework for operationalizing trustworthy AI

We propose a framework organized around five interdependent domains: decision-centered design, human-AI role delineation, failure visibility, embedded governance, and continuous evaluation. Together, these domains translate high-level trust principles into operational practices.

### 4.1 Decision-centered design

Trustworthy AI systems should be designed around the *decisions* they are intended to influence, rather than around predictive tasks alone. This requires explicit articulation of the decision context, including:

1. The clinical or operational decision to be supported
2. The user responsible for the decision
3. The expected relationship between AI output and human judgment

Without this clarity, AI systems risk becoming either prescriptive tools that override human expertise or advisory tools that are routinely ignored (7).

Decision-centered design also requires alignment with organizational incentives and constraints. For example, an AI system designed to reduce length of stay may conflict with clinician priorities related to patient safety unless decision logic and accountability are explicitly defined.

### 4.2 Human-AI role delineation

Ambiguity regarding human and AI roles is a common source of implementation failure. Trustworthy deployment requires clear delineation of tasks that are automated, tasks that are augmented, and tasks that remain exclusively human.

Role delineation should be stable and visible. Frequent, unexplained changes in AI behavior or scope can erode clinician confidence and increase cognitive burden. Importantly, delineation should include explicit override mechanisms, enabling clinicians to contest AI recommendations without penalty.

This approach mitigates automation bias while preserving the potential benefits of decision support.

### 4.3 Failure visibility and safe degradation

Healthcare environments are characterized by uncertainty, incomplete data, and evolving conditions. Trustworthy AI systems must therefore anticipate failure rather than assume perfect performance.

Failure visibility mechanisms include confidence scores, uncertainty estimates, and alerts for out-of-distribution inputs (4). When performance degrades, systems should default to safe modes that preserve human control rather than continuing to generate misleading outputs.

Opacity during failure is particularly damaging to trust. Systems that fail silently may continue to influence decisions long after their reliability has diminished.

### 4.4 Embedded governance and accountability

Operational trustworthiness depends on governance structures that define responsibility across the AI lifecycle. Governance should encompass:

- Pre-deployment risk assessment and ethical review
- Clear ownership of deployed models
- Processes for monitoring performance, bias, and drift
- Authority to modify, suspend, or retire systems when necessary

The European Commission's AI evidence pathway emphasizes that governance must be continuous and evidence-driven, rather than episodic or compliance-focused (6).

Embedding governance into routine operations ensures that trustworthiness is actively maintained rather than retrospectively audited.

### 4.5 Continuous evaluation beyond accuracy

Post-deployment evaluation should extend beyond traditional performance metrics to capture system-level impact. Relevant measures include:

- User adoption and override rates
- Effects on clinician workload and cognitive load
- Downstream clinical and operational outcomes
- Differential effects across demographic and socioeconomic groups

Such evaluation aligns with the FUTURE-AI emphasis on deployability and real-world impact, recognizing that accuracy alone is insufficient to justify continued use

### 4.6 The decision-governed trustworthy AI (DG-TAI) framework

**Figure 1:**
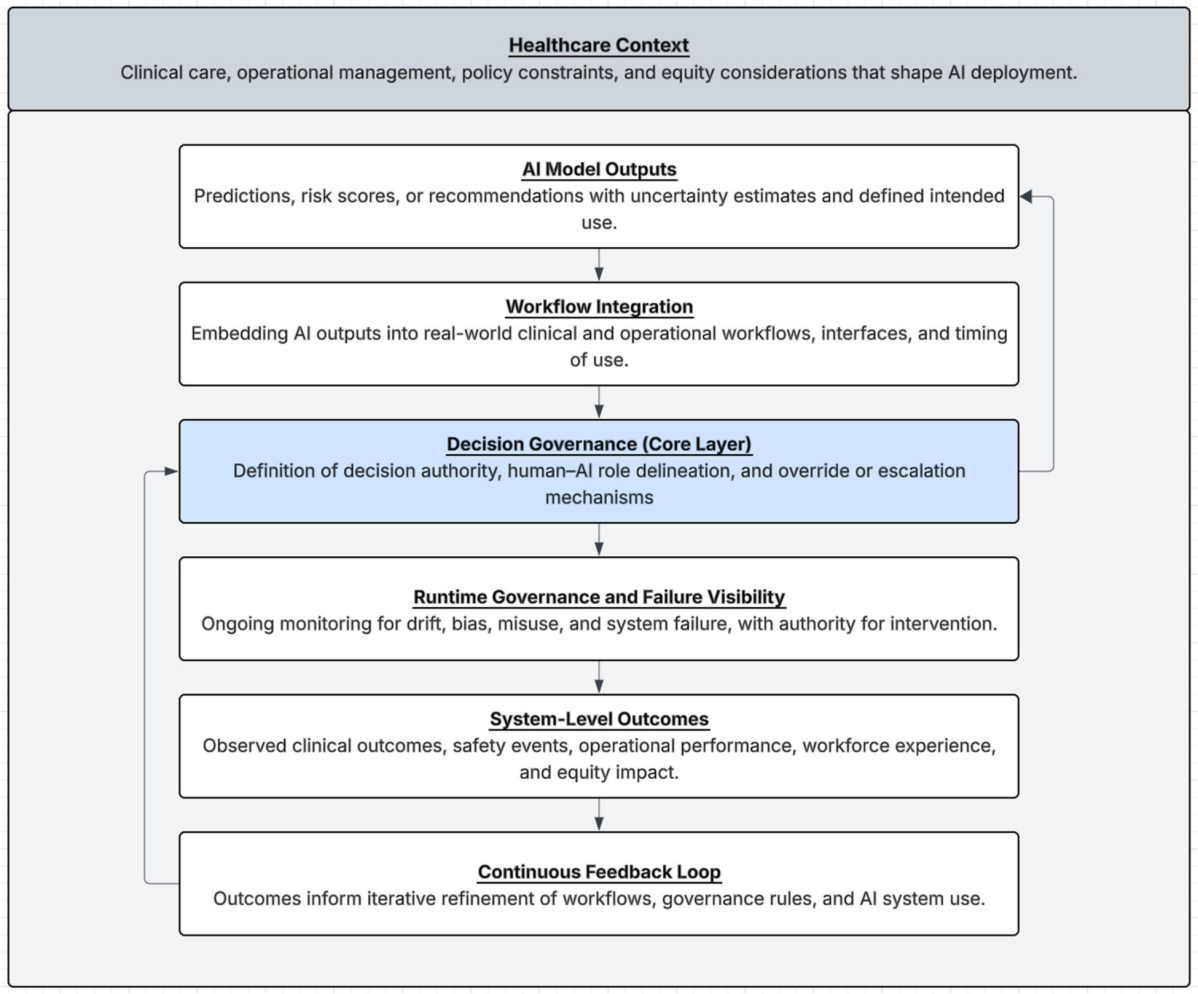
Conceptual architecture of the decision-governed trustworthy AI (DG-TAI) framework. The framework positions clinical and operational decisions not algorithmic predictions as the central locus of trust. AI model outputs are embedded within workflows that define decision authority, human–AI role delineation, and override mechanisms. Trustworthiness emerges at runtime through governance structures that ensure failure visibility, accountability, and continuous performance evaluation across clinical, operational, and equity dimensions.

To consolidate the operational principles described above, we refer to the proposed model as the Decision-Governed Trustworthy AI (DG-TAI) framework (Figure 1). The naming is intentional and descriptive rather than proprietary. It reflects the central premise that trustworthiness in healthcare AI is governed not at the level of models or predictions, but at the level of *decisions* specifically, how algorithmic outputs are interpreted, acted upon, overridden, and monitored within real-world clinical and operational workflows.

The DG-TAI framework does not introduce new ethical principles or technical requirements. Instead, it integrates established trustworthiness constructs into a decision-centric governance architecture that emphasizes accountability, failure visibility, and longitudinal oversight. In doing so, it provides a unifying structure that links abstract trust principles to operational mechanisms that can be implemented, evaluated, and adapted over time.

This framing is particularly relevant in healthcare settings, where AI systems rarely operate autonomously and instead shape decisions distributed across clinicians, administrators, and multidisciplinary teams. By making decision governance the organizing unit of analysis, the DG-TAI framework addresses a persistent gap between model validation and real-world system behavior.

### 4.7 Conceptual architecture of the DG-TAI framework

As illustrated in Figure 1, the DG-TAI framework is organized around five interdependent layers: model outputs, workflow integration, decision execution, governance mechanisms, and observed outcomes.

At the foundational layer, AI models generate predictions or recommendations. These outputs are not treated as decisions in themselves, but as inputs to structured workflows that define how information is surfaced to users, what actions are permissible, and where discretion resides. This distinction is critical: while models produce estimates, responsibility for action remains human and institutionally governed.

The workflow layer specifies human AI role delineation, including which decisions are automated, augmented, or reserved exclusively for human judgment. Importantly, this layer also defines override pathways and escalation mechanisms, ensuring that AI systems support rather than constrain.

Decisions executed within workflows then interact with governance structures that operate continuously rather than episodically. These structures include performance monitoring, bias surveillance, incident reporting, and authority to intervene when systems deviate from expected behavior. Governance is thus embedded into routine operations rather than treated as external compliance.

Finally, outcomes such as clinical, operational, experiential, and equity-related feed back into governance processes, enabling iterative refinement. Trustworthiness is therefore conceptualized as a dynamic system property that is produced and maintained through ongoing interaction between technology, humans, and institutions.

## 5 Trustworthy AI in operational workflows

While much of the trustworthy AI literature focuses on clinical decision support, operational AI systems such as those used for staffing, scheduling, or capacity management, pose comparable risks. These systems influence care indirectly by shaping resource availability and prioritization, yet their effects may be less visible to frontline clinicians.

Operational AI systems require the same trust principles as clinical tools, with particular emphasis on transparency and governance. Because operational decisions often affect large populations, small biases or errors can produce systemic inequities if left unchecked (1).

Embedding trustworthy AI into operational workflows therefore requires cross-functional governance and explicit mechanisms for contestability and oversight.

## 6 Implications for health systems and policy

Operationalizing trustworthy AI demands organizational investment beyond technical development. Health systems must cultivate interdisciplinary expertise spanning informatics, clinical leadership, operations, and ethics. Training programs should emphasize not only how AI works, but how it should be questioned, monitored, and governed.

For policymakers and regulators, the framework suggests a shift from static pre-approval criteria toward evidence of real-world performance and governance maturity. Trustworthiness should be evaluated longitudinally, with attention to how AI systems behave under changing conditions.

Importantly, trustworthy AI should be framed not as a constraint on innovation but as an enabler of scale. Systems that fail transparently and are governed responsibly are more likely to earn sustained trust and adoption.

## 7 Discussion

This paper advances a socio-technical framework for operationalizing trustworthy artificial intelligence (AI) in healthcare by shifting emphasis from algorithmic properties to system-level behavior within clinical and operational workflows. While existing guidelines articulate important ethical and technical principles, their translation into practice remains inconsistent. The framework proposed here addresses this gap by identifying operational mechanisms through which trustworthiness can be enacted, observed, and sustained over time.

A central contribution of this work is the explicit reframing of trustworthiness as an emergent property rather than a static attribute. This perspective aligns with empirical observations that AI systems may perform well in development environments yet fail to generate value or even introduce risk; once deployed in real-world settings. By foregrounding workflow integration, decision authority, and governance, the framework highlights dimensions of AI performance that are often invisible in traditional validation studies but critical to real-world success.

### 7.1 Implications for clinical practice

For clinicians, the framework underscores the importance of clarity in how AI systems intersect with professional judgment. Ambiguity regarding whether AI outputs are advisory, suggestive, or prescriptive has been shown to undermine trust and contribute to both over-reliance and disengagement. Decision-centered design and explicit human-AI role delineation help mitigate these risks by aligning AI recommendations with clinicians’ mental models and accountability structures.

The emphasis on failure visibility is particularly salient in clinical contexts, where uncertainty is inherent and stakes are high. Systems that explicitly communicate confidence and limitations may initially appear less decisive, yet they are more likely to foster durable trust. Importantly, such transparency supports appropriate skepticism rather than blind reliance, reinforcing professional autonomy rather than displacing it.

### 7.2 Implications for operational and administrative AI

Operational AI systems such as those used for staffing, scheduling, capacity management, and resource allocation present distinct trust challenges that are often underappreciated. Unlike clinical decision support tools, operational systems frequently exert indirect influence on patient care, making their effects less visible and their failures harder to detect. The framework's emphasis on embedded governance and continuous evaluation is therefore particularly relevant in operational domains.

By extending trustworthy AI principles beyond bedside decision-making, this work contributes to a more comprehensive understanding of how AI shapes healthcare delivery at scale. Operational trustworthiness requires not only technical robustness but also transparency, contestability, and alignment with organizational values, particularly with respect to equity and workforce well-being.

### 7.3 Governance, accountability, and organizational maturity

A recurring theme in this framework is the centrality of governance. Trustworthy AI cannot be sustained through *ad hoc* oversight or *post hoc* audits alone. Instead, it requires clearly defined ownership, escalation pathways, and authority to intervene when systems underperform or behave unexpectedly. These requirements imply a level of organizational maturity that many health systems are still developing.

From this perspective, trustworthiness is as much an institutional capability as it is a technical one. Health systems that lack data governance infrastructure, interdisciplinary oversight committees, or mechanisms for continuous monitoring may struggle to operationalize trustworthy AI, regardless of model quality. Conversely, organizations that invest in governance capacity may be better positioned to deploy AI safely and at scale.

### 7.4 Equity and unintended consequences

Equity considerations cut across all dimensions of trustworthy AI. While fairness is often framed as a statistical property of models, inequities may also arise from workflow design, differential access to AI-enabled services, or uneven adoption across clinical settings. The framework's focus on system-level evaluation encourages attention to these downstream effects.

Continuous monitoring of differential outcomes across demographic and socioeconomic groups is therefore essential. Importantly, such monitoring should not be limited to clinical outcomes alone but should also encompass operational consequences, such as differential wait times, staffing burdens, or access to specialized services. By embedding equity considerations into routine evaluation, health systems can move beyond compliance toward proactive risk mitigation.

### 7.5 Limitations and future research

This work is conceptual in nature and does not present empirical validation of the proposed framework. While the framework is grounded in existing literature and consensus guidance, its practical utility will depend on context-specific implementation and empirical testing. Future research should examine how different elements of the framework influence trust, adoption, and outcomes across diverse healthcare settings.

Potential research directions include comparative studies of governance models, evaluations of failure visibility mechanisms, and longitudinal analyses of trust dynamics following AI deployment. Additionally, there is a need for validated metrics that capture workflow-level trustworthiness, complementing existing technical performance measures.

### 7.6 Conceptual contribution and positioning relative to prior frameworks

The World Health Organization (1) guidance establishes a foundational ethical and human-rights framework for artificial intelligence in health, emphasizing principles such as transparency, accountability, equity, and protection of human autonomy. However, it intentionally remains high-level and does not prescribe concrete operational mechanisms for implementation, monitoring, or value realization. The FUTURE-AI framework (8) advances the field by translating trustworthy AI into six core principles and 30 consensus-based best practices across the AI lifecycle, offering substantially more practical guidance for healthcare AI development and deployment. Nevertheless, FUTURE-AI focuses primarily on system-level practices and stops short of defining enterprise governance structures, decision rights, or performance management mechanisms. In contrast, the DG-TAI framework extends both WHO and FUTURE-AI by functioning as an execution-oriented governance operating model that embeds trustworthy AI principles into organizational structures, lifecycle controls, and measurable outcomes. DG-TAI explicitly links ethics and trustworthiness to accountability, continuous monitoring, and value realization, enabling health systems to operationalize trustworthy AI at scale rather than treating it solely as a design-time or compliance consideration.

While numerous frameworks have articulated principles for trustworthy or responsible AI, the DG-TAI framework contributes to the literature in three distinct ways.

First, it repositions trustworthiness as a runtime system property rather than a design-time attribute. Existing frameworks largely emphasize pre-deployment validation and ethical compliance. In contrast, DG-TAI foregrounds post-deployment behavior, recognizing that trust is shaped by how systems perform under real-world conditions, including uncertainty, drift, and organizational change.

Second, the framework elevates decisions as the primary unit of governance, rather than models or predictions. This shift has practical and ethical significance. Decisions are observable, auditable, and attributable, making them more amenable to governance than abstract model properties. By anchoring trustworthiness in decision execution, the framework aligns AI oversight with established clinical and administrative accountability structures.

Third, DG-TAI explicitly extends trustworthy AI considerations to operational workflows, including staffing, scheduling, capacity management, and resource allocation. While prior work has focused predominantly on clinical decision support, operational AI systems often exert broader systemic influence and may generate inequities that are less visible but equally consequential. The framework treats clinical and operational AI symmetrically, applying the same trust, governance, and evaluation principles across both domains.

Importantly, the contribution of this work lies not in introducing new ethical norms, but in integrating existing principles into a coherent operational architecture. This synthesis enables health systems to move from aspirational trustworthiness to implementable, monitorable practice.

### 7.7 Implications for evaluation and future research

The DG-TAI framework suggests several directions for future empirical research. First, it motivates the development of metrics that assess trustworthiness at the decision and workflow levels, complementing traditional model-centric performance measures. Second, it invites comparative evaluation of governance models, examining how different ownership and escalation structures influence trust, adoption, and outcomes. Third, it highlights the need for longitudinal studies that examine how trust evolves over time as AI systems and organizational contexts change.

By offering a decision-governed lens, the framework provides a foundation for testable hypotheses without presupposing specific technologies or use cases. As such, it is designed to remain applicable across clinical domains, operational functions, and health system contexts.

### 7.8 Operationalizing DG-TAI in practice: a pilot-to-scale use case

Consider a large, multi-hospital healthcare system seeks to deploy an AI-enabled workforce forecasting and staffing optimization solution to address persistent nurse shortages, reduce reliance on premium labor, and improve patient safety. Applying the DG-TAI framework, the organization begins by establishing a formal governance structure that defines decision rights and accountability across HR, clinical operations, IT, data governance, and compliance. Furthermore, Executive sponsors, model owners, data stewards, and a trust or ethics review function are identified to oversee workforce equity, clinician well-being, and regulatory considerations.

Rather than proceeding directly to enterprise-wide rollout, DG-TAI requires deployment as a controlled pilot within a limited set of units or facilities. During the design and pre-deployment phase, historical staffing, census, acuity, and scheduling data supporting the pilot are subjected to rigorous data quality, representativeness, and bias assessments. The model is evaluated for differential impact across shifts, units, and demographic proxies, and technical risks such as robustness and generalizability are assessed in the context of the pilot environment. Advancement beyond the pilot is contingent on meeting predefined trust and performance thresholds, not solely on predictive accuracy.

During the pilot phase, DG-TAI operationalizes continuous monitoring through a defined set of key performance indicators spanning technical, ethical, and operational dimensions. These include forecast accuracy and drift, override and exception rates, staffing equity indicators, overtime hours, vacancy coverage, and clinician experience proxies. Governance forums review these metrics at regular intervals, enabling rapid identification of unintended consequences and informed decision-making about model refinement.

Only after the pilot demonstrates sustained performance, acceptable equity impacts, and operational value does DG-TAI authorize scaled deployment across additional units or the broader enterprise. Post-scale, monitoring and governance mechanisms remain active, with formal escalation and remediation pathways triggered by threshold breaches or contextual changes. Insights from the pilot and early scale phases are systematically captured and fed back into the organization's AI maturity model, strengthening future implementations. This pilot-to-scale approach illustrates how DG-TAI operationalizes trustworthy AI as a phased, accountable process, bridging ethical intent, real-world execution, and sustained value realization in complex healthcare settings.

## 8 Conclusion

As artificial intelligence becomes increasingly embedded in healthcare delivery, the question of trust has emerged as a defining challenge. This paper argues that trustworthy AI cannot be reduced to a checklist of model attributes or ethical principles. Instead, trustworthiness must be understood as a dynamic, system-level achievement that depends on how AI is integrated into clinical and operational workflows, how responsibilities are allocated, and how failures are anticipated and managed.

By proposing a framework that operationalizes trustworthiness through decision-centered design, human–AI role delineation, failure visibility, embedded governance, and continuous evaluation, this work offers a pragmatic pathway from principle to practice. The framework does not seek to replace existing ethical or technical guidance but to complement it by addressing the operational realities of AI deployment in healthcare.

Ultimately, the sustainability of AI in healthcare will depend not on whether systems are merely accurate or innovative, but on whether they are reliable, transparent, and aligned with professional values and organizational goals. Health systems that invest in the socio-technical foundations of trustworthy AI may be better positioned to realize its potential while minimizing unintended harm. In this sense, trustworthiness should be viewed not as a constraint on innovation, but as a prerequisite for scale, resilience, and long-term impact.

Future research should empirically test elements of the framework, examining how different governance models, role delineations, or evaluation strategies influence trust, adoption, and outcomes across settings.
